# Retraction Note: Vitamin D alleviates cognitive dysfunction and brain damage induced by copper sulfate intake in experimental rats: focus on its combination with donepezil

**DOI:** 10.1007/s00210-023-02690-4

**Published:** 2023-08-29

**Authors:** Mohamed M. Elseweidy, Mohamed Mahrous, Sousou I. Ali, Mohamed A. Shaheen, Nahla N. Younis

**Affiliations:** 1 Department of Biochemistry, Faculty of Pharmacy, Zagazig University, Zagazig, 44519 Egypt; 2 Department of Biochemistry, Faculty of Pharmacy, Port-Said University, Port-Said, 42526 Egypt; 3 Department of Histology and Cell Biology, Faculty of Human Medicine, Zagazig University, Zagazig, 44519 Egypt

**Retraction Note: Naunyn–Schmiedeberg's Archives of Pharmacology (2023) 396:1931–1942**

**https://doi.org/10.1007/s00210-023–02449-x**

The Editor-in-Chief has retracted this article. After publication, concerns were raised regarding some of the images in the figures. Specifically:

Figure 5c and e appear to overlap with rotation.

In Fig. 7a, b, d and e, the images appear to contain repeated patterns.

The authors have provided the raw data to address these concerns; however, the same issues were observed in the original images. The Editor-in-Chief therefore no longer has confidence in the presented data.

Sousou I. Ali and Nahla N. Younis do not agree to this retraction. Mohamed A. Shaheen, Mohamed Mahrous and Mohamed M. Elseweidy have not explicitly stated whether they agree to this retraction.

